# Supplementary material for: Phytochemical Screening and Bioactive Properties of Juglans regia L. Pollen
Source: Antioxidants (Basel). 2022 Oct 18;11(10):2046. doi: 10.3390/antiox11102046 (PMC9598064; doi:10.3390/antiox11102046)
Supplement: Supplementary file 1 [file antioxidants-11-02046-s001.zip › S1-Chromatogram.pdf]

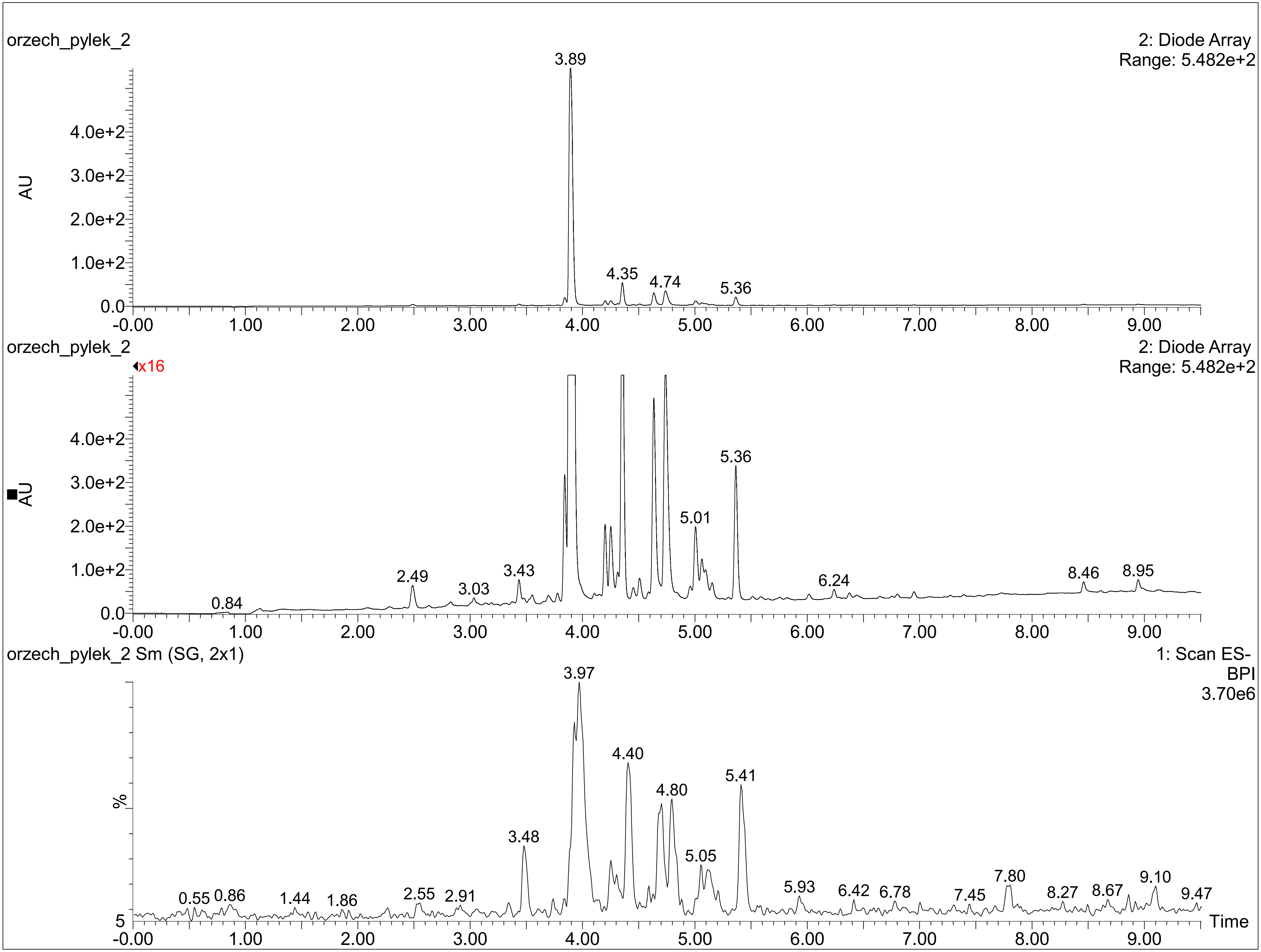
orzech_pylek_2
2: Diode Array
Range: 5.482e+2
3.89
4.35
4.74
5.36
AU
4.0e+2
3.0e+2
2.0e+2
1.0e+2
0.0
-0.00
1.00
2.00
3.00
4.00
5.00
6.00
7.00
8.00
9.00
orzech_pylek_2
2: Diode Array
Range: 5.482e+2
x16
5.36
5.01
2.49
3.03
3.43
0.84
6.24
8.46
8.95
orzech_pylek_2 Sm (SG, 2x1)
1: Scan ES-
BPI
3.70e6
3.97
4.40
4.80
5.41
3.48
5.05
0.55
0.86
1.44
1.86
2.55
2.91
5.93
6.42
6.78
7.45
7.80
8.27
8.67
9.10
9.47
%
5
Time
