# Supplementary material for: Phytochemical Screening and Bioactive Properties of Juglans regia L. Pollen
Source: Antioxidants (Basel). 2022 Oct 18;11(10):2046. doi: 10.3390/antiox11102046 (PMC9598064; doi:10.3390/antiox11102046)
Supplement: Supplementary file 1 [file antioxidants-11-02046-s001.zip › Supplementary Tables.pdf]

antioxidants

*Supplementary material*

# Phytochemical screening and bioactive properties of *Juglans regia* L. pollen

**Natalia Żurek, Karolina Pycia, Agata Pawłowska and Ireneusz Tomasz Kapusta ***

Department Food Technology and Human Nutrition, Institute of Food Technology and Nutrition, University of Rzeszow, 4 Zelwerowicza St., 35-601 Rzeszow, Poland
* Correspondence: ikapusta@ur.edu.pl; Tel.: +48-17-785-5238

**Content:**

**Supplementary Table S1:** Correlation between TP, TF, compounds present in the highest concentration in *J. regia* pollen extract and antioxidant activity.

**Supplementary Table S2:** Correlation between TP, TF, compounds present in the highest concentration in *J. regia* pollen extract and antiproliferation activity.

UPLC chromatogram of J. regia pollen extracts and UV-Vis and MS spectra of identified compounds.

**Table S1**. Correlation between TP, TF, compounds present in the highest concentration (quercetin 3-*O*-sophoroside, kaempferol 3-*O*-sophoroside, dimethoxyflavone-7-*O*-beta-D-glucoside) in *J. regia* pollen extract and antioxidant activity determined by ABTS, CUPRAC, ChA, $O_2^{\bullet -}$, and $OH^-$ methods.

| | **TP** | **TF** | **Quercetin 3-*O*-sophoroside** | **Kaempferol 3-*O*-sophoroside** | **dimethoxyflavone-7-*O*-beta-D-glucoside** |
|---|---|---|---|---|---|
| **ABTS** | 0.992* | 0.681 | 0.998* | 0.999** | 0.873 |
| **CUPRAC** | 0.634 | 0.998* | -0.476 | -0.537 | -0.486 |
| **ChA** | -0.999** | 0.931 | -0.987 | -0.988 | -0.704 |
| **$O_2^{\bullet -}$** | 0.513 | 0.507 | 0.666 | 0.622 | 0.596 |
| **$OH^-$** | -0.683 | -0.687 | -0.533 | -0.508 | -0.519 |

(+) positive correlation, (-) negative correlation, *significant differences at $p < 0.05$, ** significant differences at $p < 0.01$.

**Table S2**. Correlation between TP, TF, compounds present in the highest concentration (quercetin 3-*O*-sophoroside, kaempferol 3-*O*-sophoroside, dimethoxyflavone-7-*O*-beta-D-glucoside) in *J. regia* pollen extract and antiproliferation activity against MCF-7, DLD-1, Caco-2, U87MG, U251MG, SK-Mel-29, and CCD841 CoN cell lines.

| | **TP** | **TF** | **Quercetin 3-*O*-sophoroside** | **Kaempferol 3-*O*-sophoroside** | **dimethoxyflavone-7-*O*-beta-D-glucoside** |
|---|---|---|---|---|---|
| **MCF-7** | -0.971 | -0.977* | -0.999* | -0.873 | -0.884 |
| **DLD-1** | 0.494 | -0.488 | -0.644 | -0.610 | -0.725 |
| **Caco-2** | -0.676 | -0.981* | -0.803 | -0.711 | -0.632 |
| **U87MG** | -0.516 | -0.590 | -0.668 | -0.769 | -0.703 |
| **U251MG** | 0.098 | 0.305 | -0.090 | -0.295 | -0.478 |
| **SK-Mel-29** | -0.358 | -0.451 | -0.528 | -0.678 | -0.749 |
| **CCD841 CoN** | -0.189 | -0.195 | -0.371 | -0.209 | -0.331 |

(+) positive correlation, (-) negative correlation, *significant differences at $p < 0.05$, ** significant differences at $p < 0.01$.
