# Supplementary figures and images for: Phytochemical Screening and Bioactive Properties of Juglans regia L. Pollen
Source: Antioxidants (Basel). 2022 Oct 18;11(10):2046. doi: 10.3390/antiox11102046 (PMC9598064; doi:10.3390/antiox11102046)

orzech\_pylek\_2 516 (4.396) Cm (511:523)

1: Scan ES-  
1.18e6

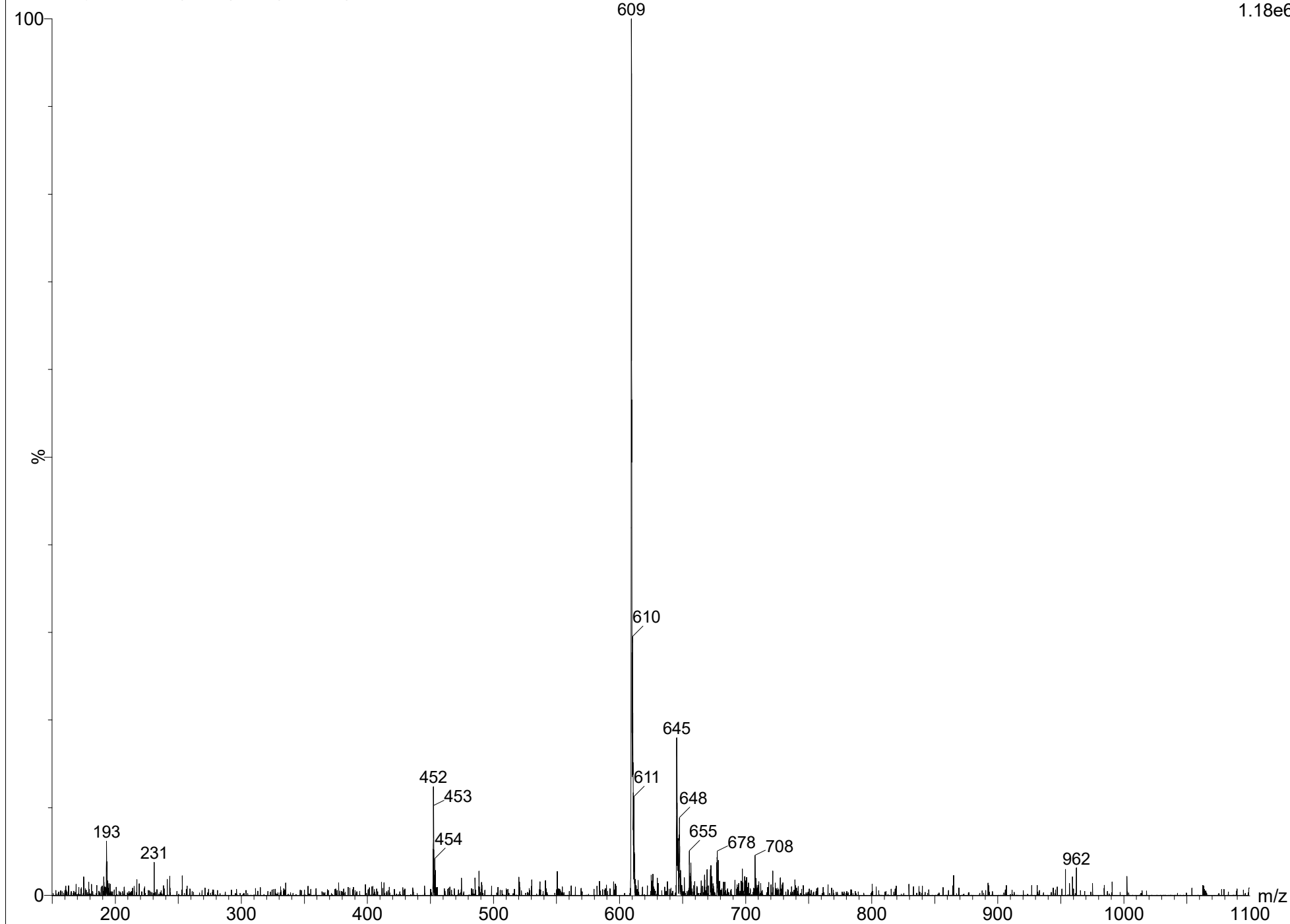

Supplement: Supplementary file 1 [file antioxidants-11-02046-s001.zip › S10-Compound_11_12_MS_Spectrum.pdf]

orzech\_pylek\_2 539 (4.592) Cm (529:540)

1: Scan ES-  
2.56e5

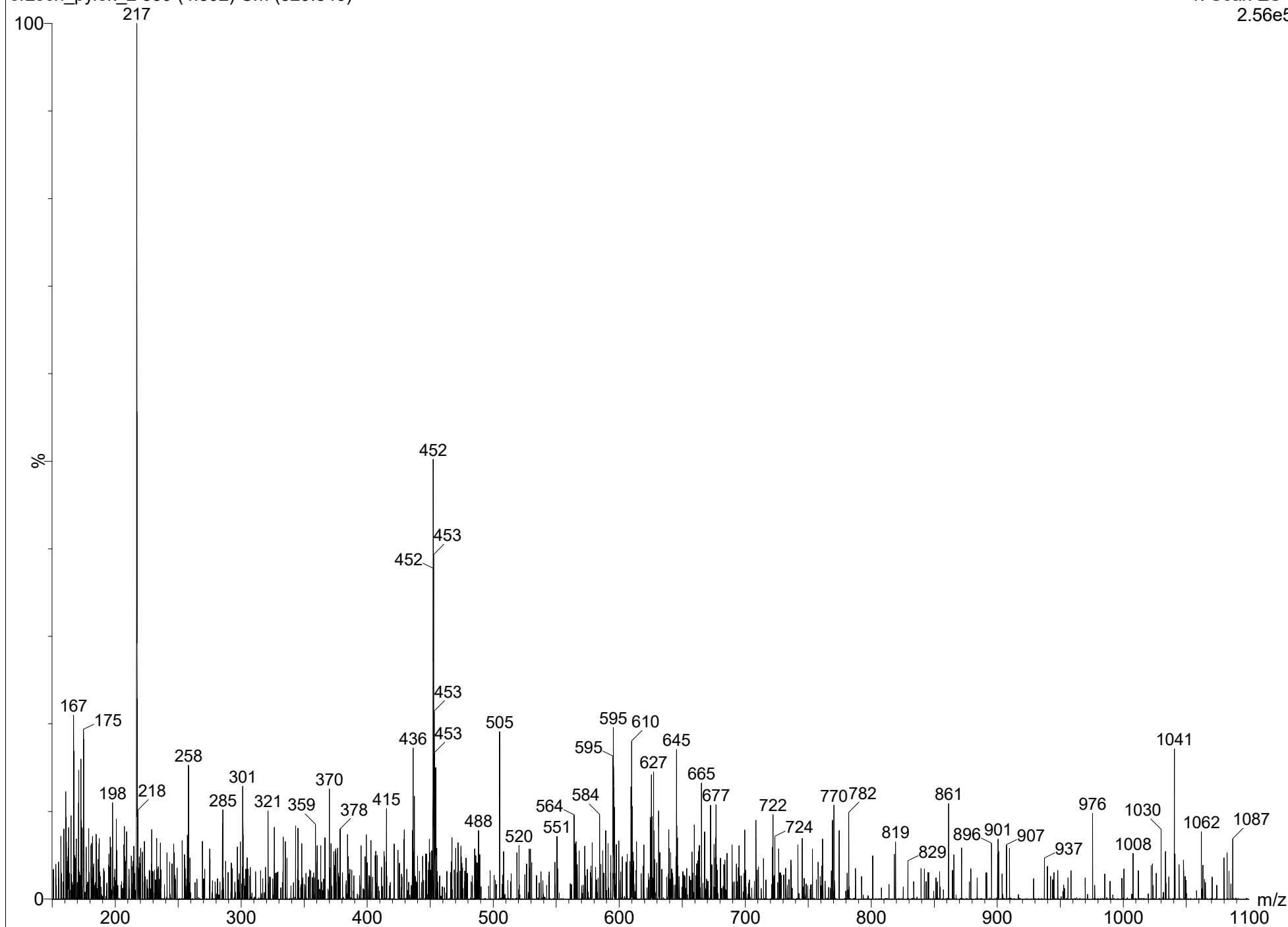

Supplement: Supplementary file 1 [file antioxidants-11-02046-s001.zip › S11-Compound_13_MS_Spectra.pdf]

orzech\_pylek\_2 549 (4.677) Cm (545:554)

1: Scan ES-  
1.11e6

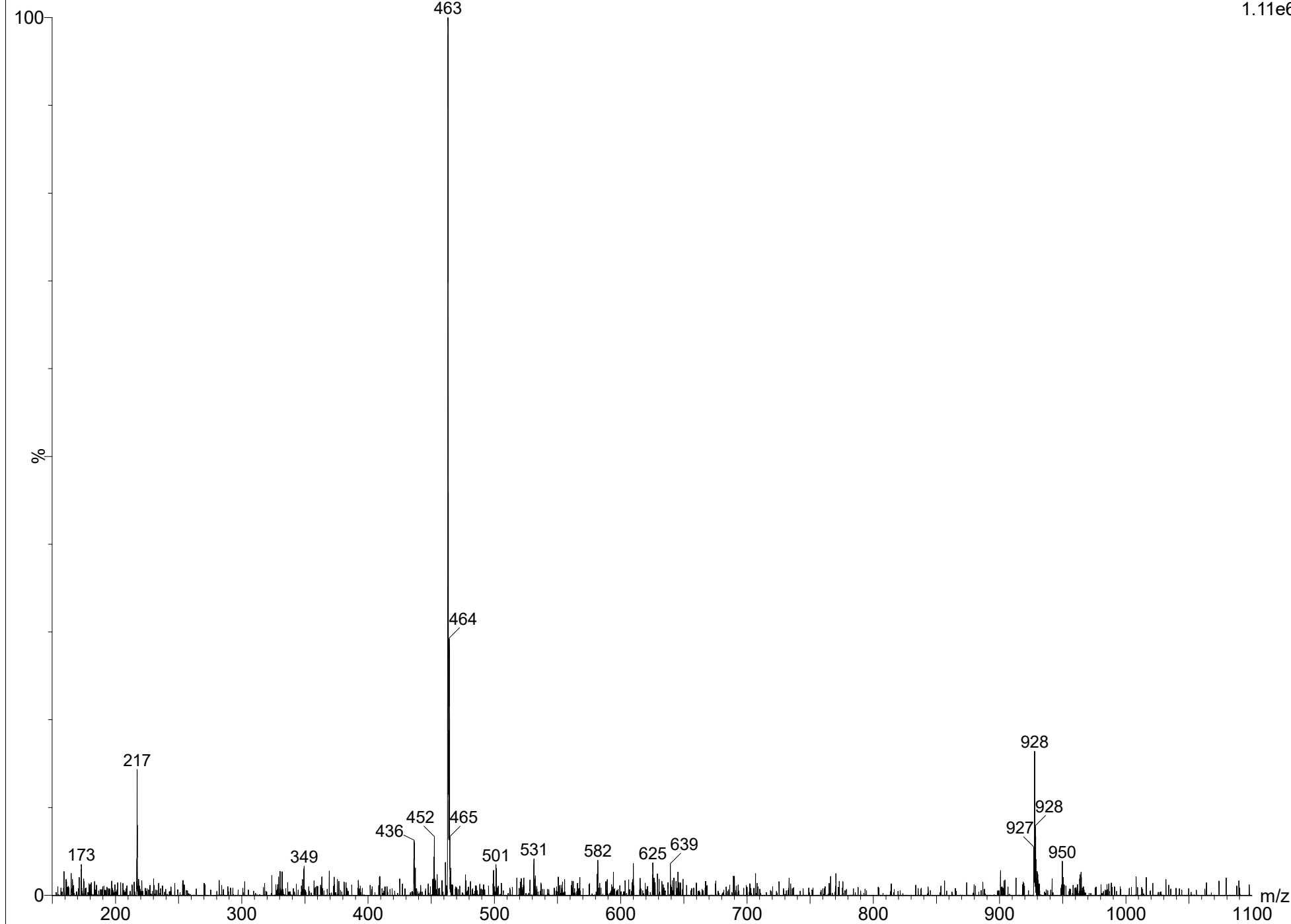

Supplement: Supplementary file 1 [file antioxidants-11-02046-s001.zip › S12-Compound_14_MS_Spectrum.pdf]

orzech\_pylek\_2 563 (4.797) Cm (557:563-560:571)

1: Scan ES-  
1.90e5

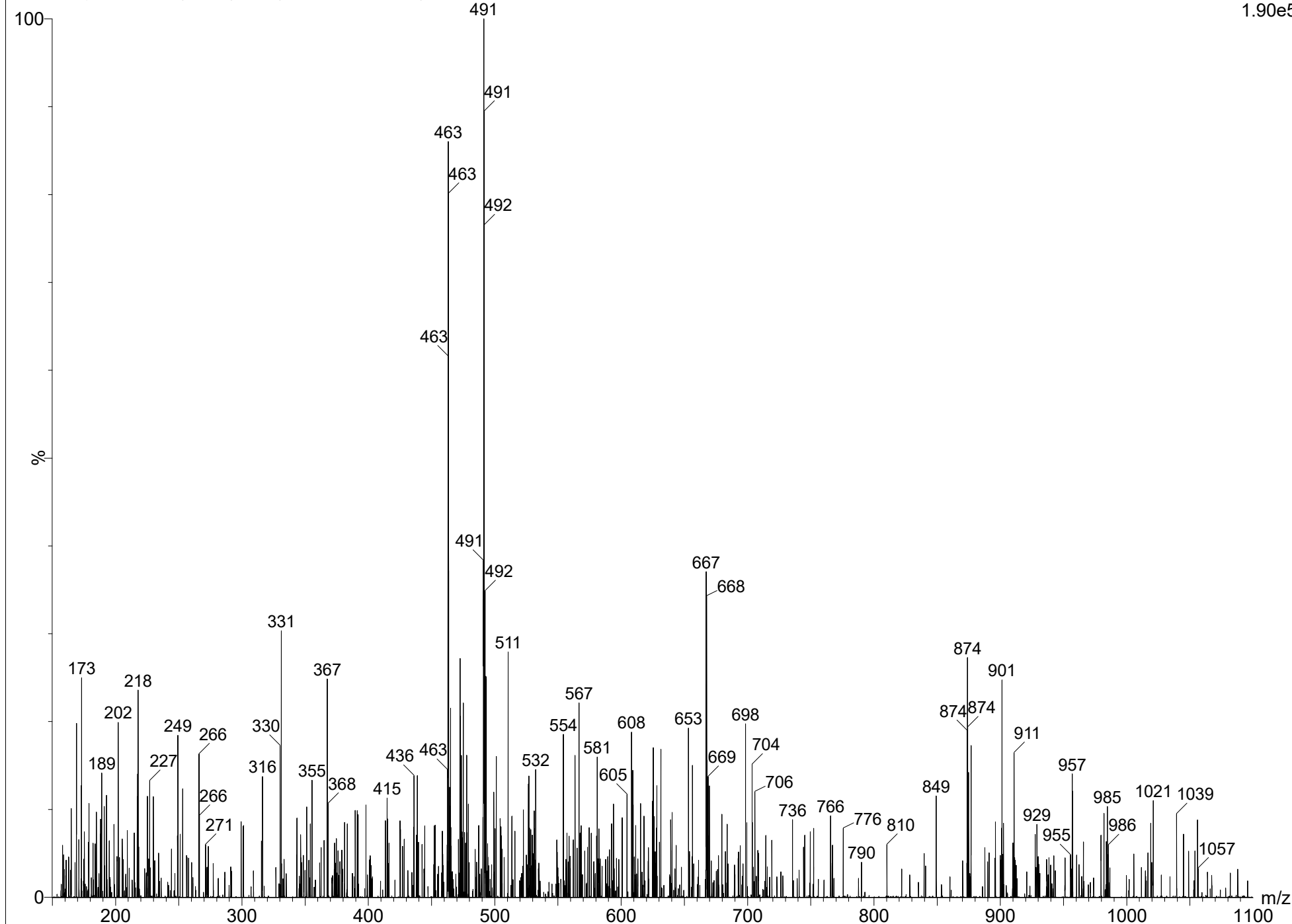

Supplement: Supplementary file 1 [file antioxidants-11-02046-s001.zip › S13-Compound_15_MS_Spectrum.pdf]

OK\_1 5693 (4.743)

2: Diode Array  
1.837

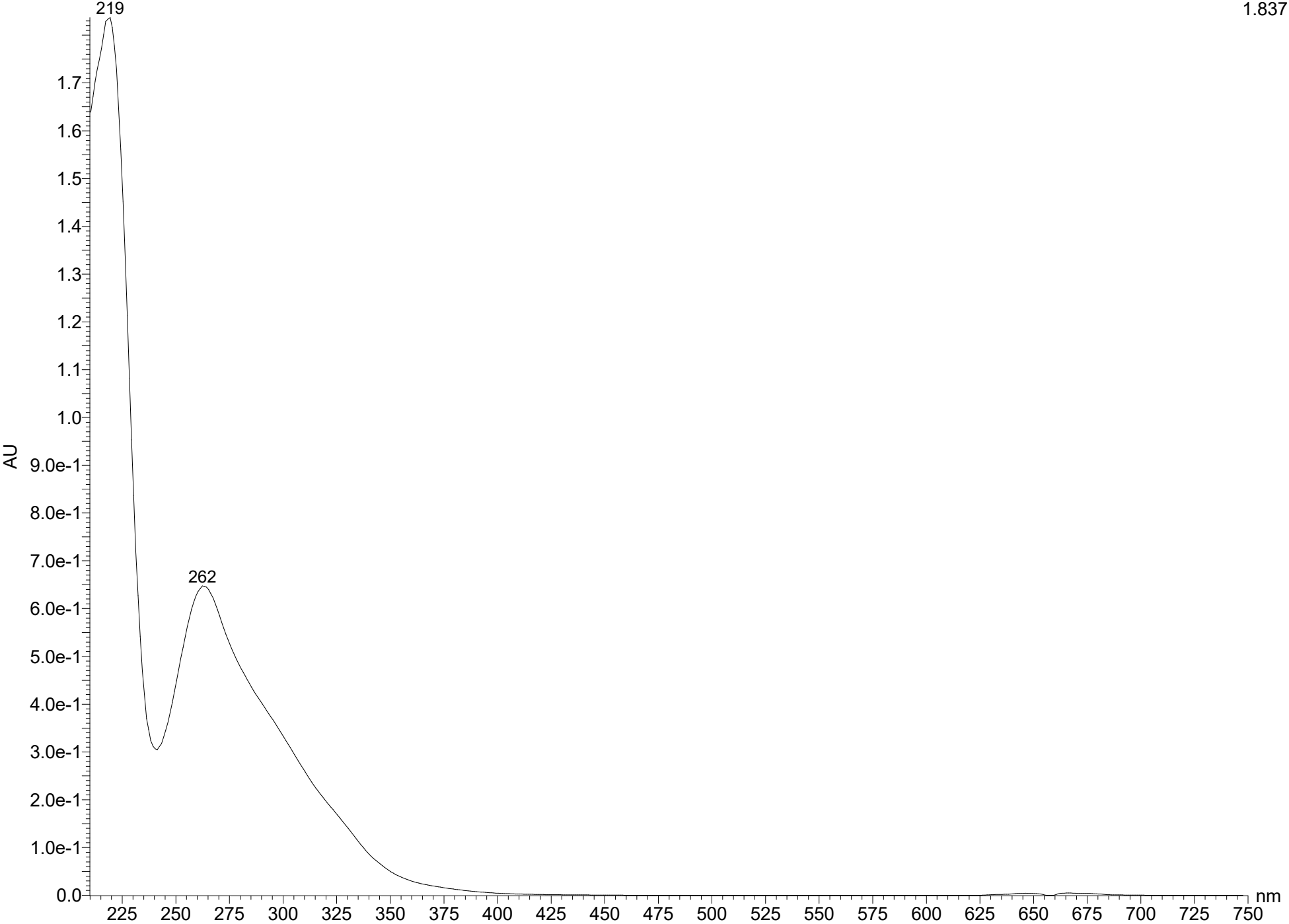

Supplement: Supplementary file 1 [file antioxidants-11-02046-s001.zip › S14-Compound_15_UV_Spectrum.pdf]

orzech\_pylek\_2 590 (5.027) Cm (583:590)

1: Scan ES-  
2.02e5

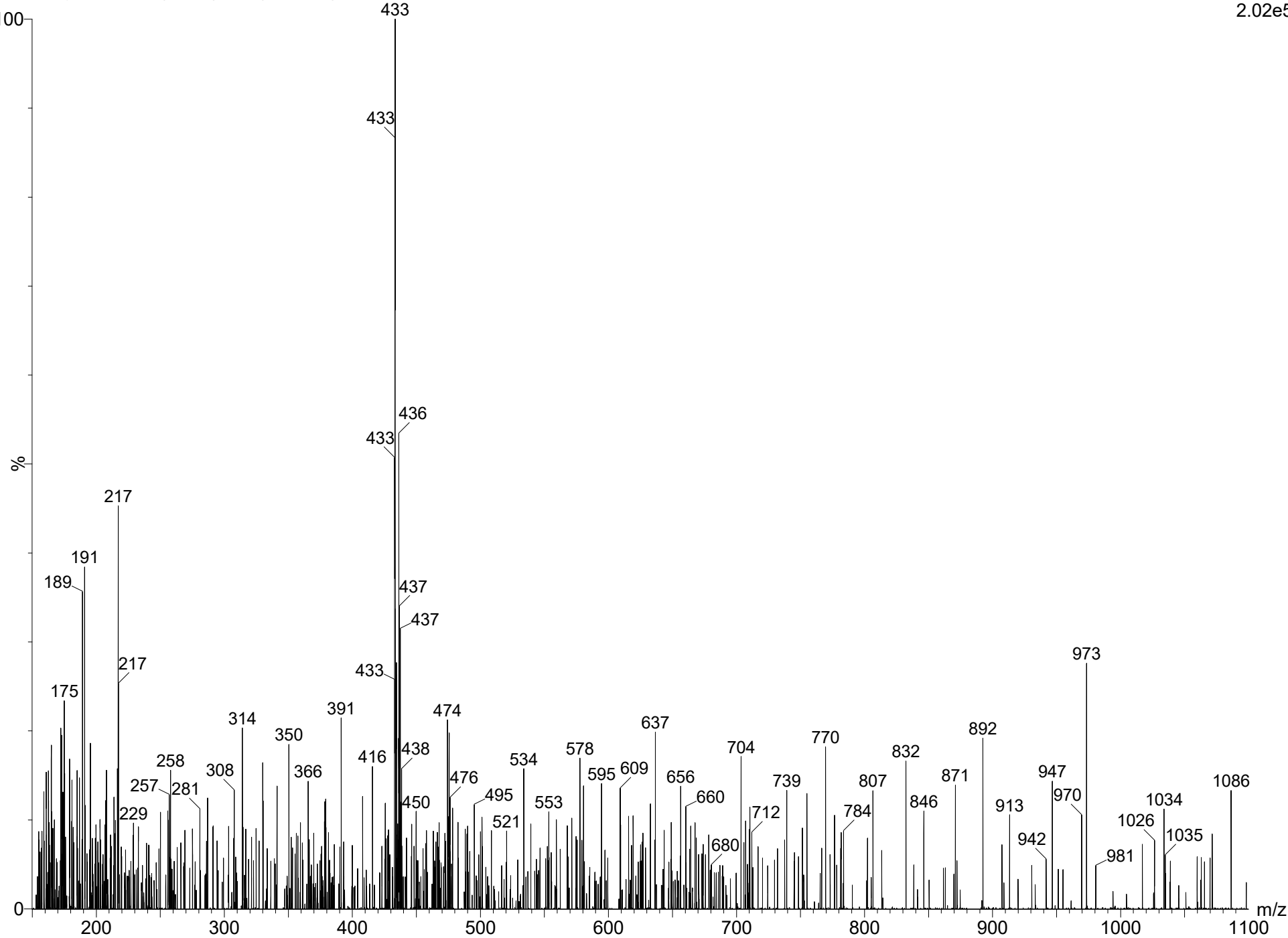

Supplement: Supplementary file 1 [file antioxidants-11-02046-s001.zip › S15-Compound_16_18_20_MS_Spectrum.pdf]

orzech\_pylek\_2 595 (5.069)

1: Scan ES-  
6.85e5

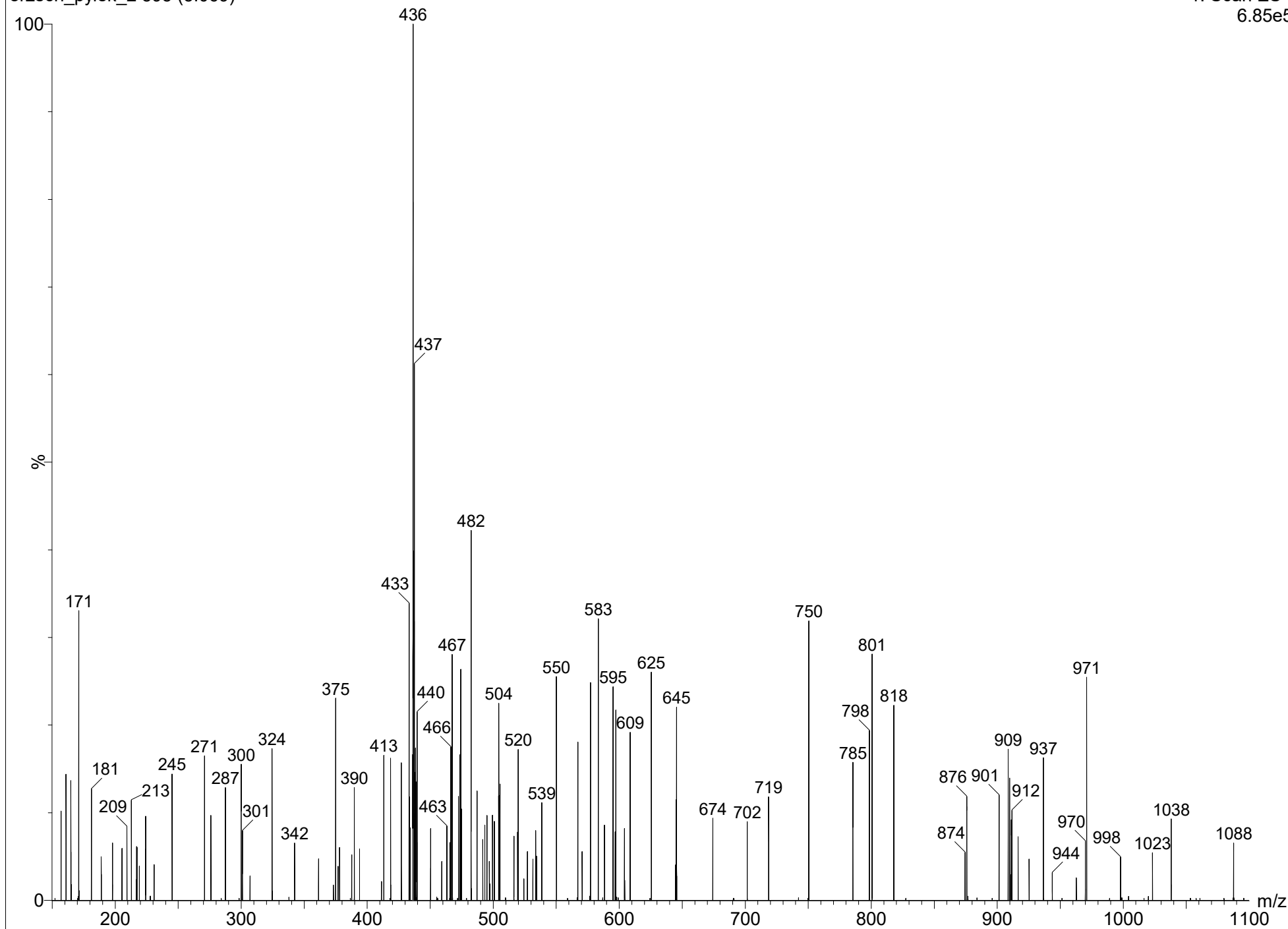

Supplement: Supplementary file 1 [file antioxidants-11-02046-s001.zip › S16-Compound_17_MS_Spectrum.pdf]

orzech\_pylek\_2 607 (5.172)

1: Scan ES-  
4.35e5

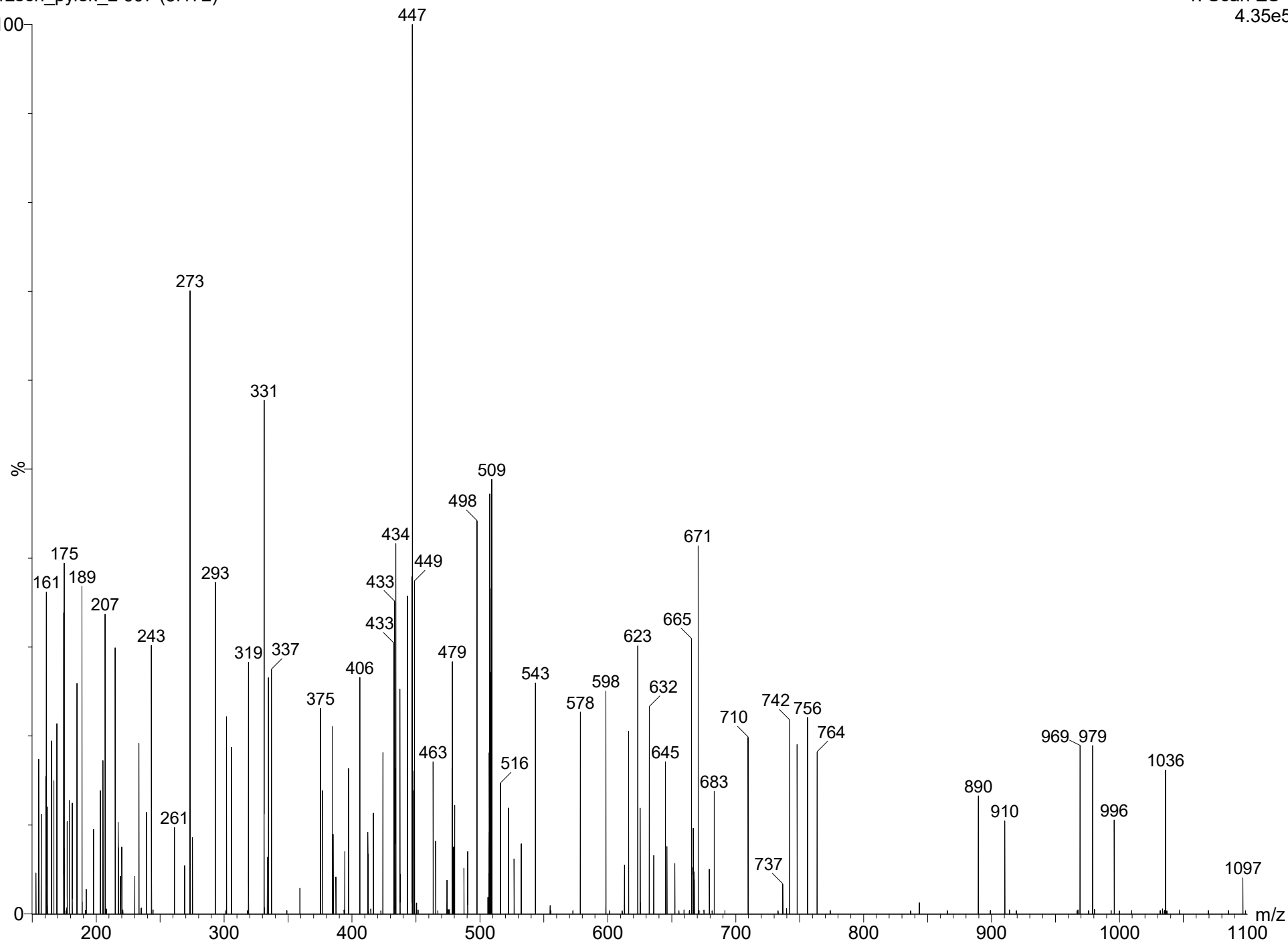

Supplement: Supplementary file 1 [file antioxidants-11-02046-s001.zip › S17-Compound_19_MS_Spectrum.pdf]

orzech\_pylek\_2 637 (5.427) Cm (633:641)

1: Scan ES-  
1.40e6

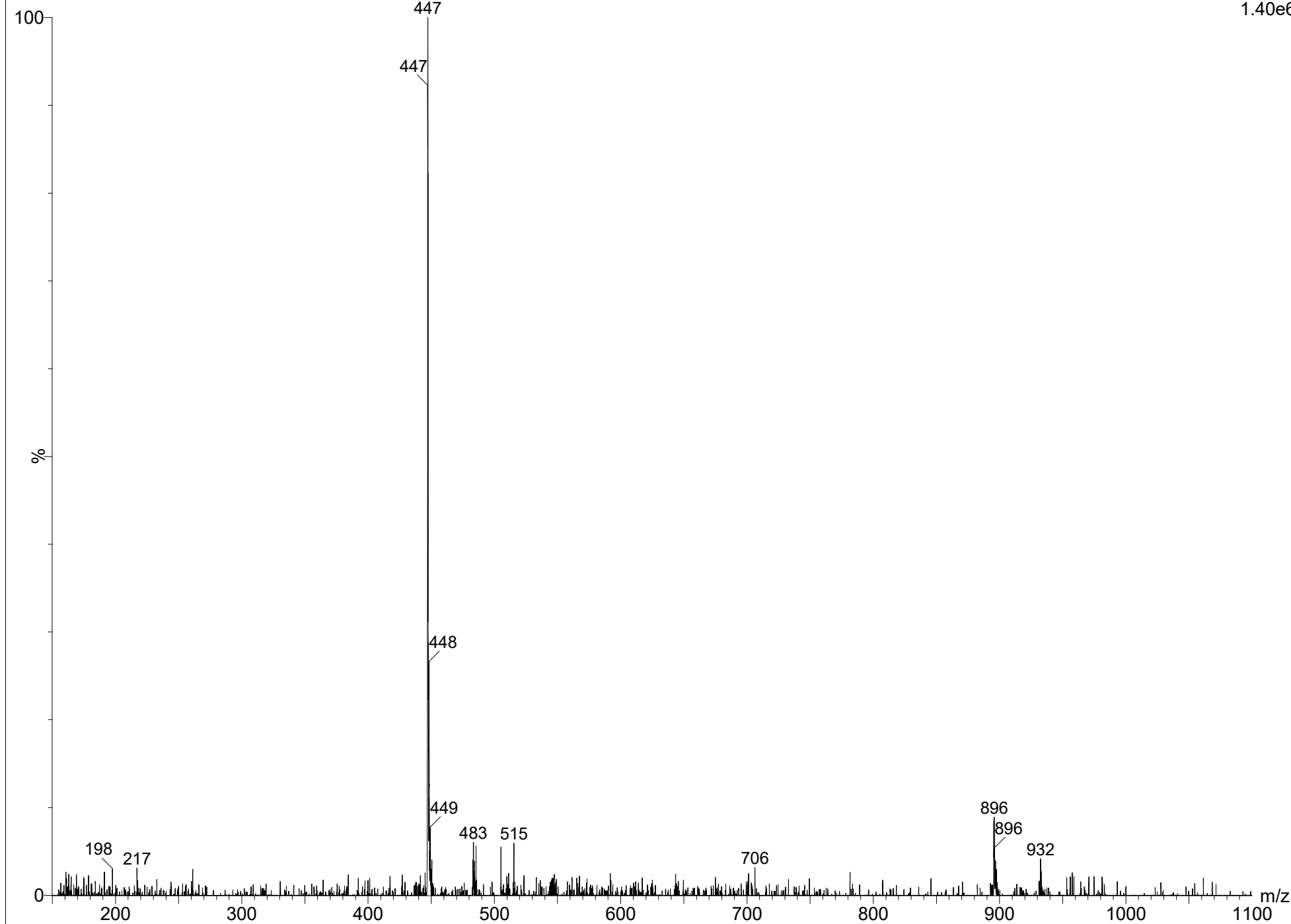

Supplement: Supplementary file 1 [file antioxidants-11-02046-s001.zip › S18-Compound_21_MS_Spectrum.pdf]

orzech\_pylek\_2 715 (6.092) Cm (710:716)

1: Scan ES-  
1.11e5

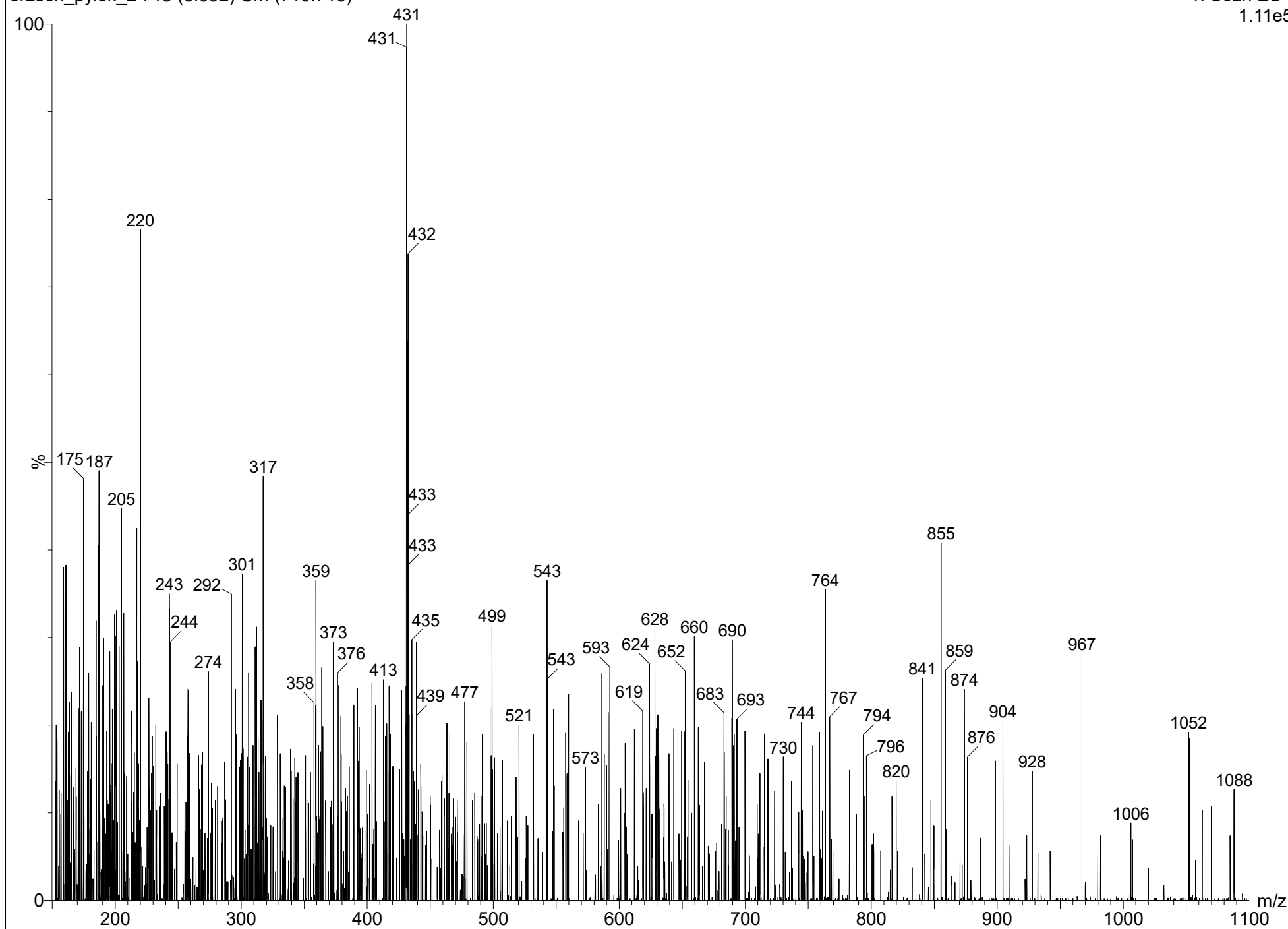

Supplement: Supplementary file 1 [file antioxidants-11-02046-s001.zip › S19-Compound_22_MS_Spectrum.pdf]

orzech\_pylek\_2 4126 (3.437)

2: Diode Array  
1.446e-1

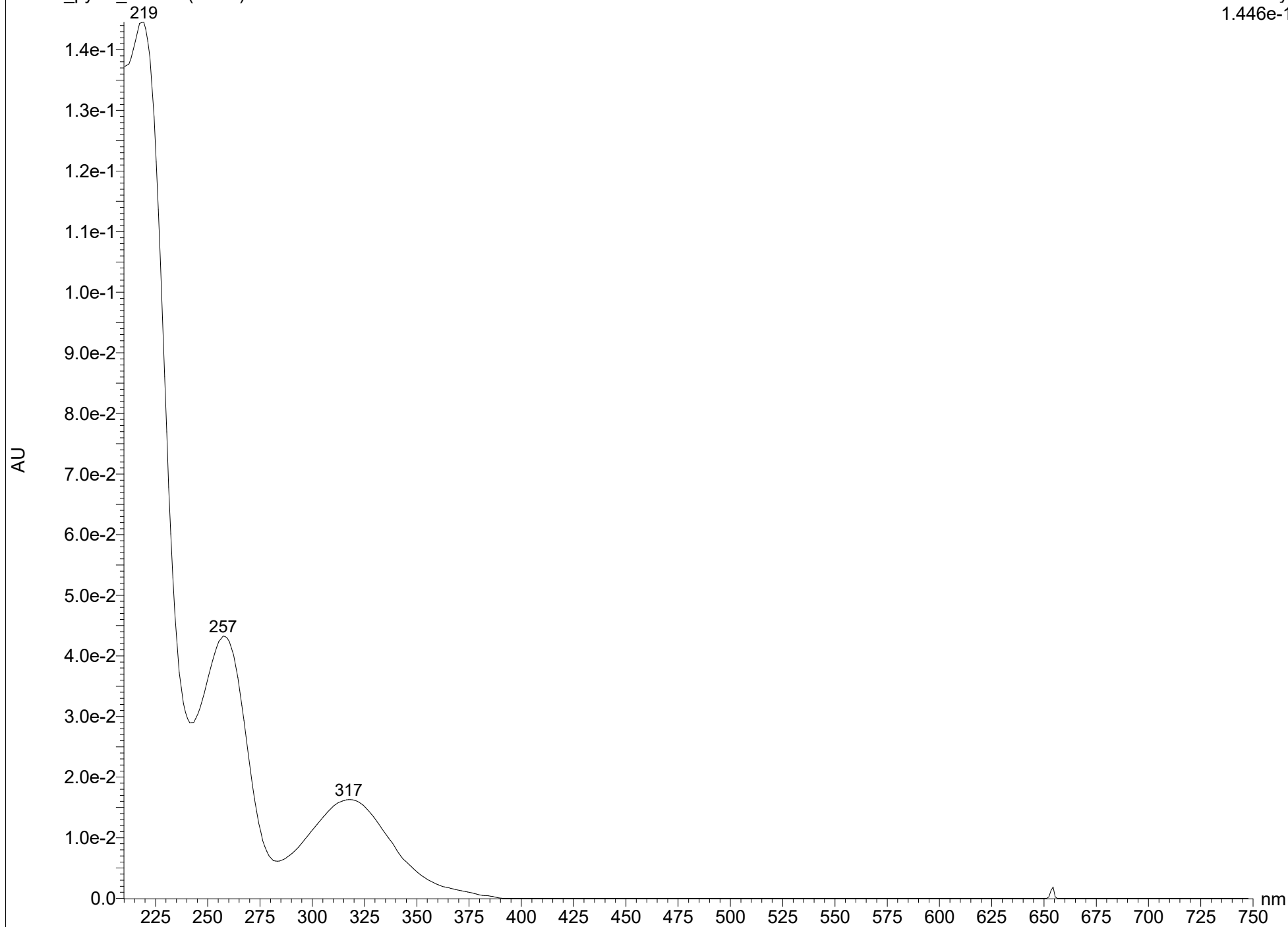

Supplement: Supplementary file 1 [file antioxidants-11-02046-s001.zip › S2-Componds 3_4_5_6_UV_Spectra.pdf]

orzech\_pylek\_2 744 (6.339) Cm (733:744)

1: Scan ES-  
7.80e4

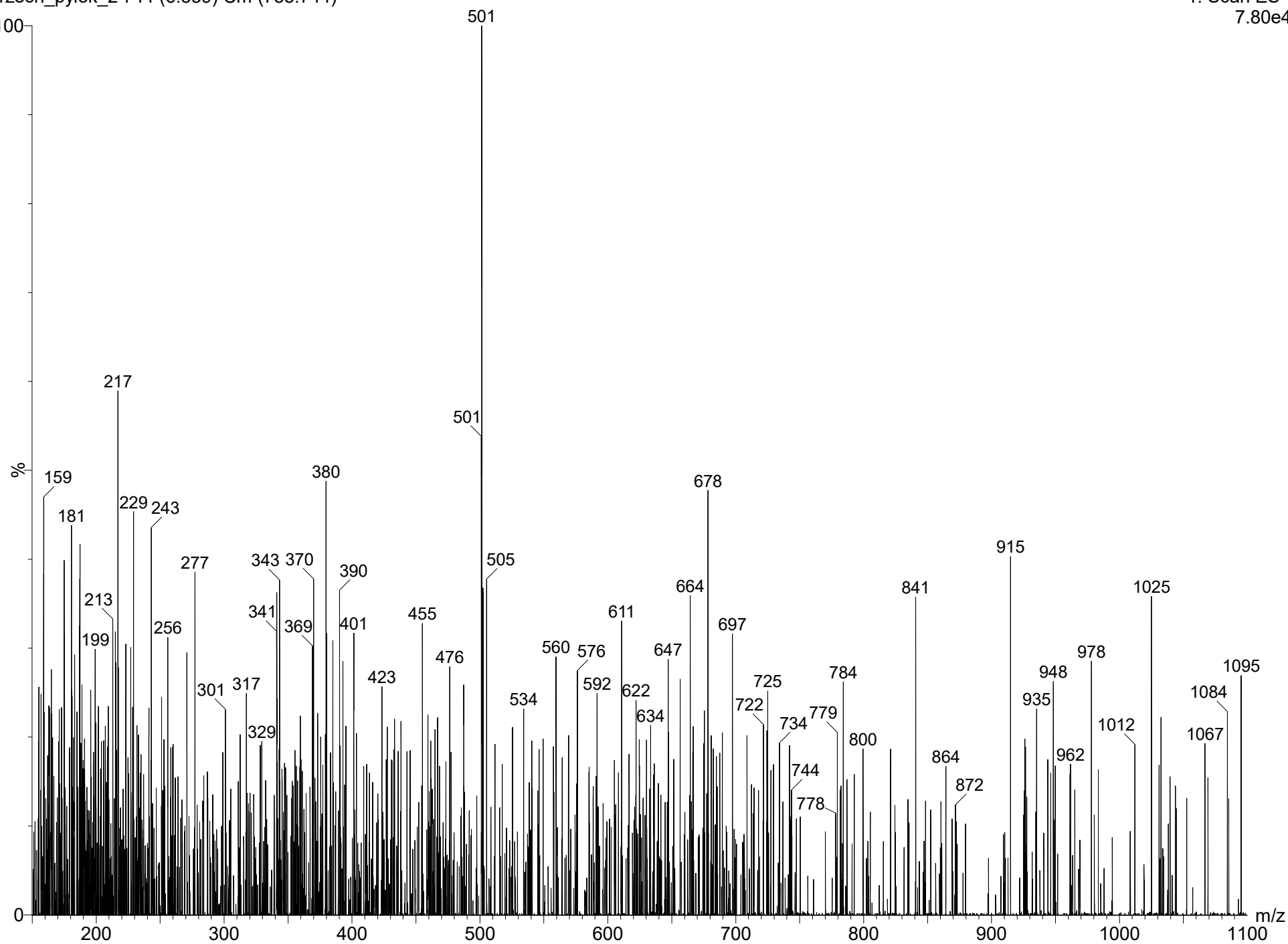

Supplement: Supplementary file 1 [file antioxidants-11-02046-s001.zip › S20-Compound_23_MS_Spectrum.pdf]

orzech\_pylek\_2 753 (6.416) Cm (750:757)

1: Scan ES-  
1.39e5

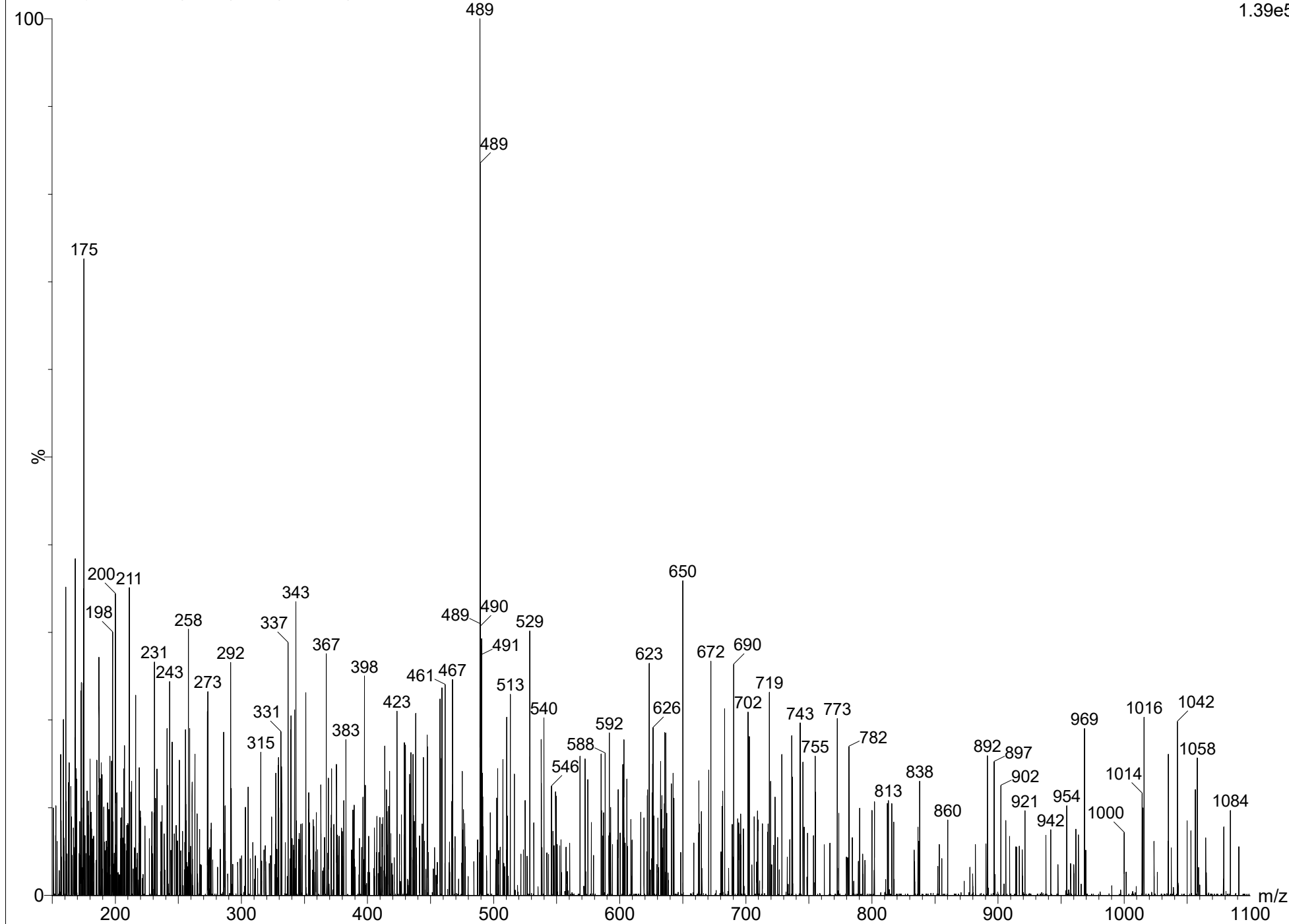

Supplement: Supplementary file 1 [file antioxidants-11-02046-s001.zip › S21-Compound_24_MS_Spectrum.pdf]

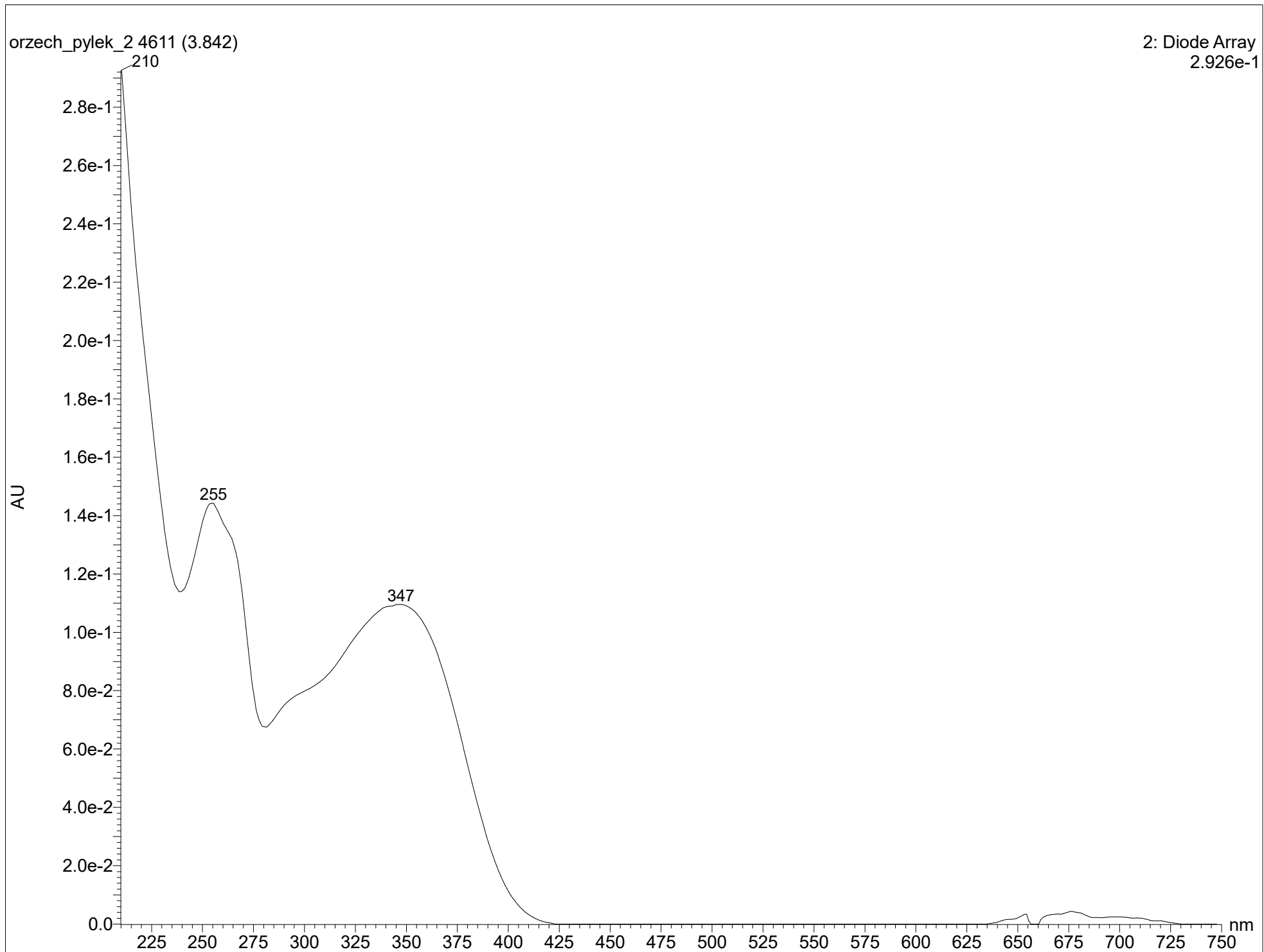

Supplement: Supplementary file 1 [file antioxidants-11-02046-s001.zip › S22-Compounds_1_7_8_9_14_16_18_20_21_UV_Spectra.pdf]

orzech\_pylek\_2 461 (3.928)

1: Scan ES-  
3.27e6

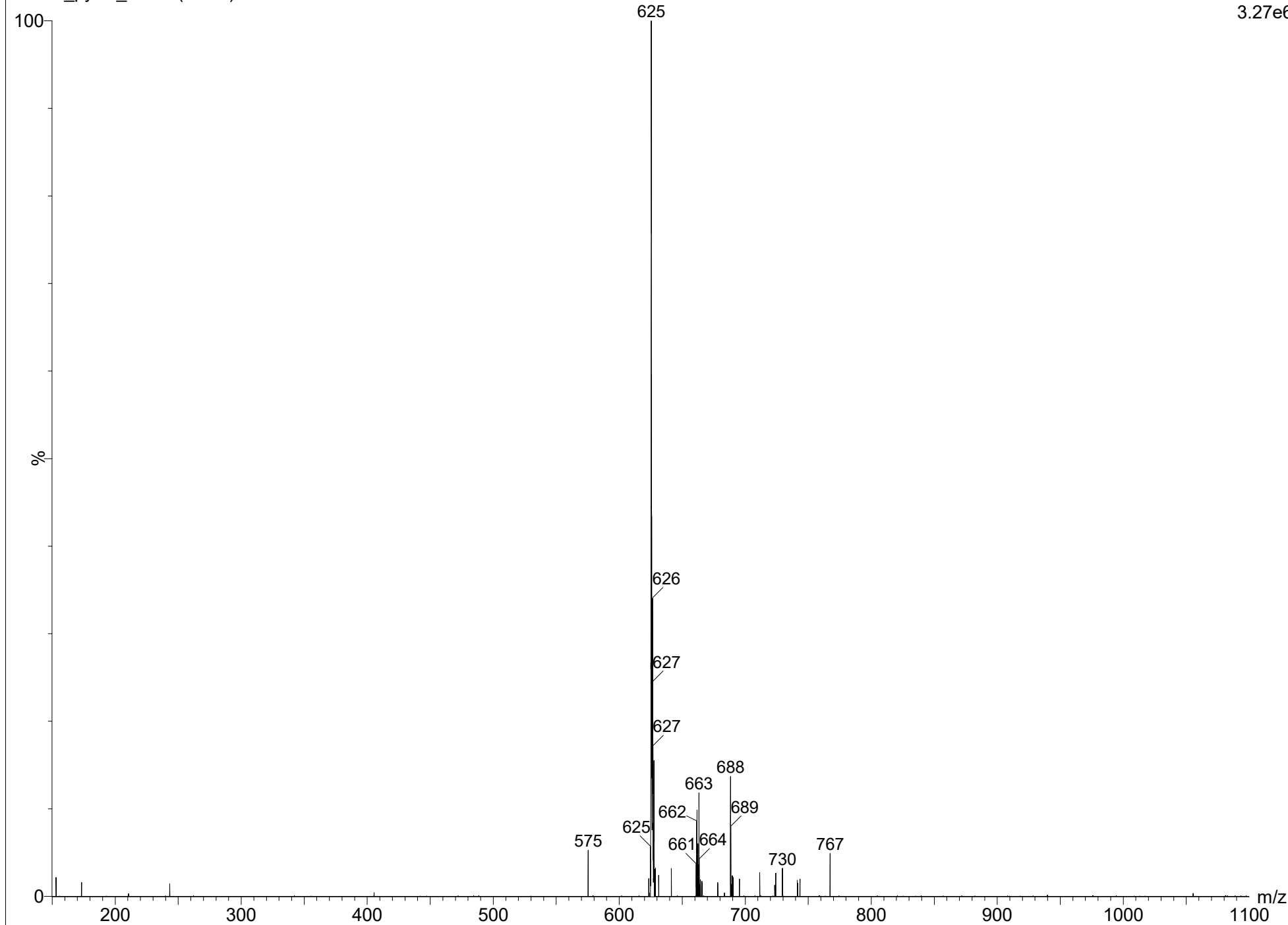

Supplement: Supplementary file 1 [file antioxidants-11-02046-s001.zip › S23-Compounds_7_8_MS_Spectrum.pdf]

orzech\_pylek\_2 5102 (4.251)

2: Diode Array  
1.638e-1

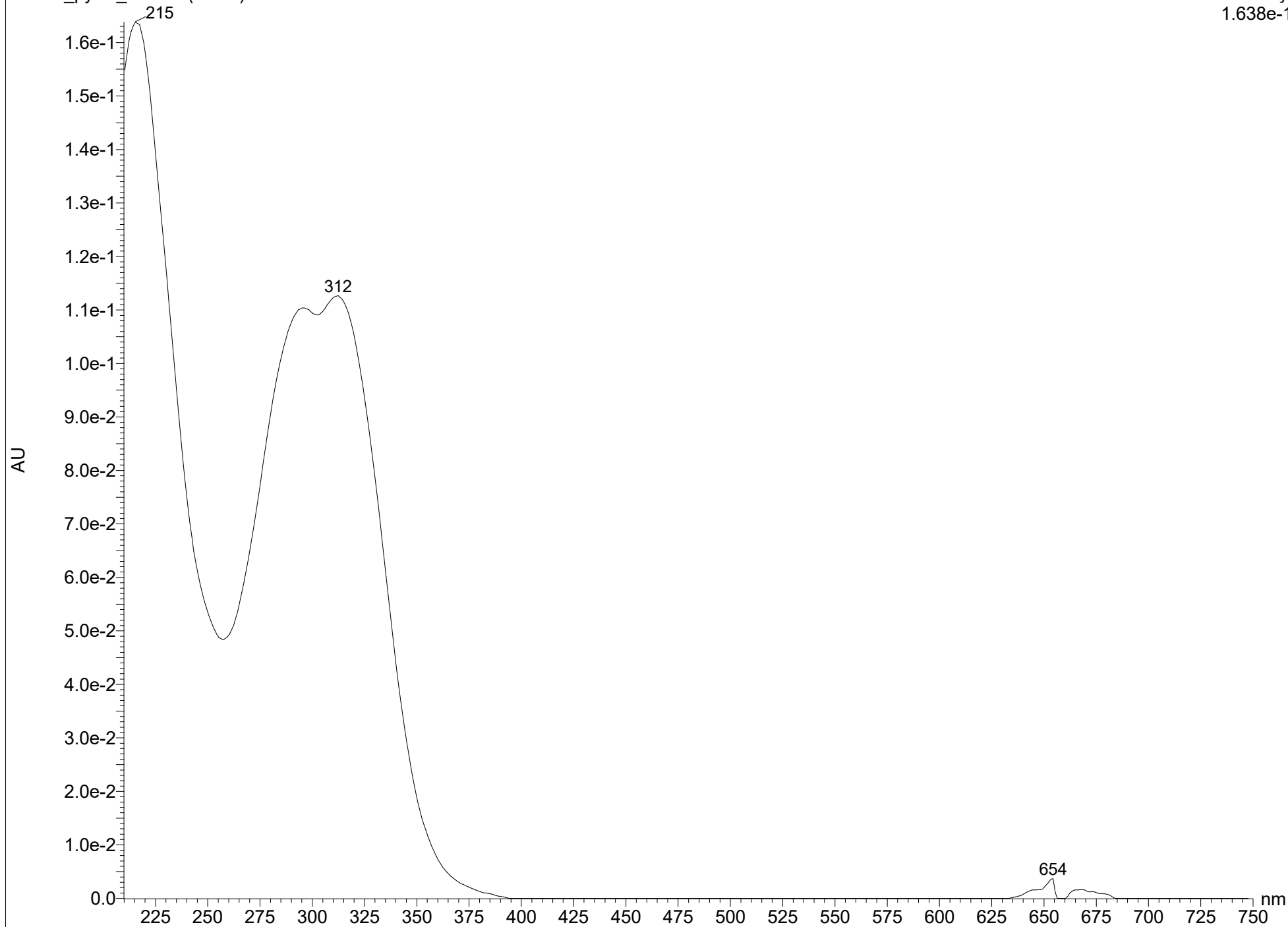

Supplement: Supplementary file 1 [file antioxidants-11-02046-s001.zip › S24-Compounds_10_13_17_UV_Spectra.pdf]

orzech\_pylek\_2 5230 (4.358)

2: Diode Array  
6.009e-1

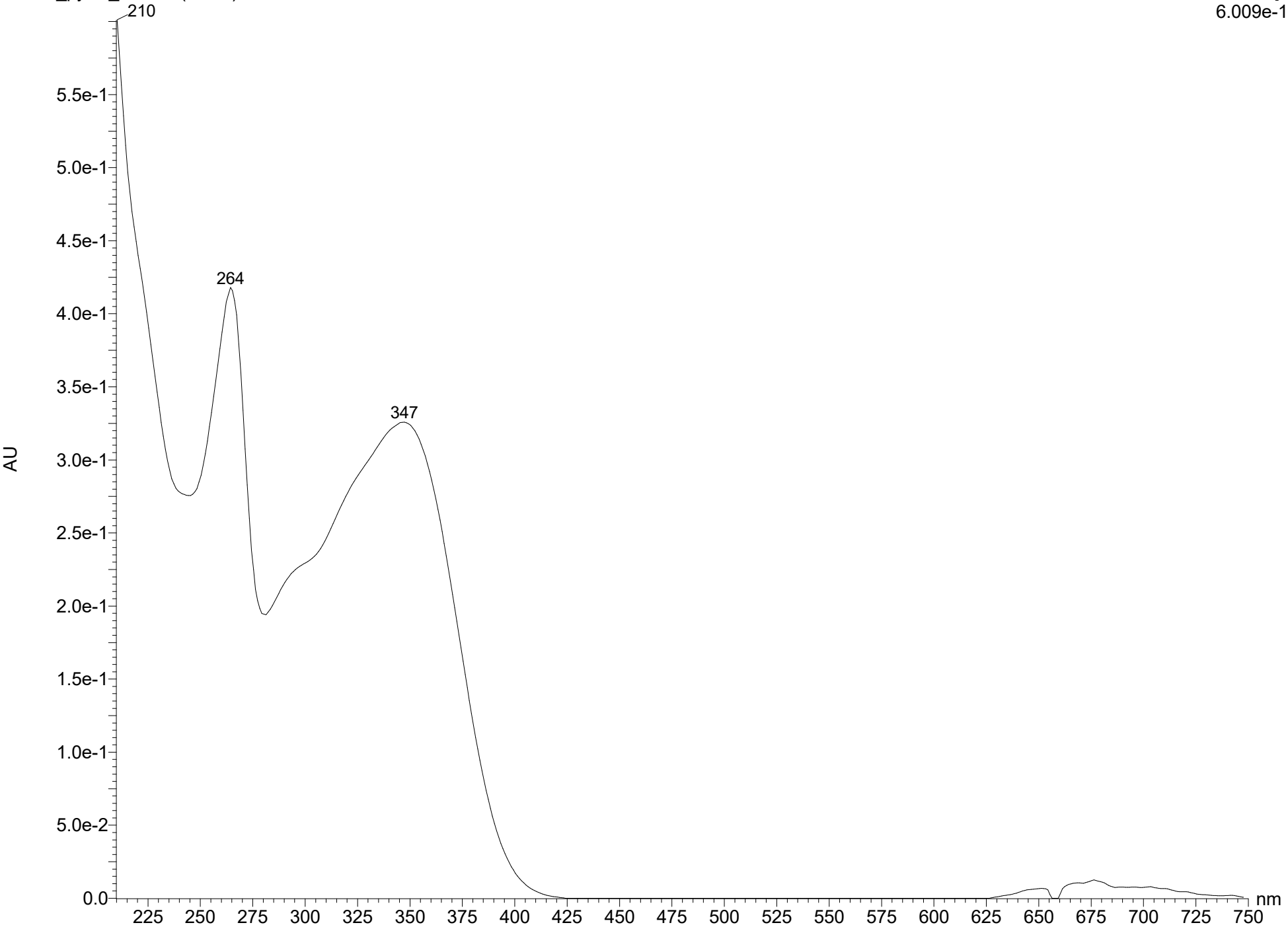

Supplement: Supplementary file 1 [file antioxidants-11-02046-s001.zip › S25-Compounds_11_12_19_22_24_UV_Spectra.pdf]

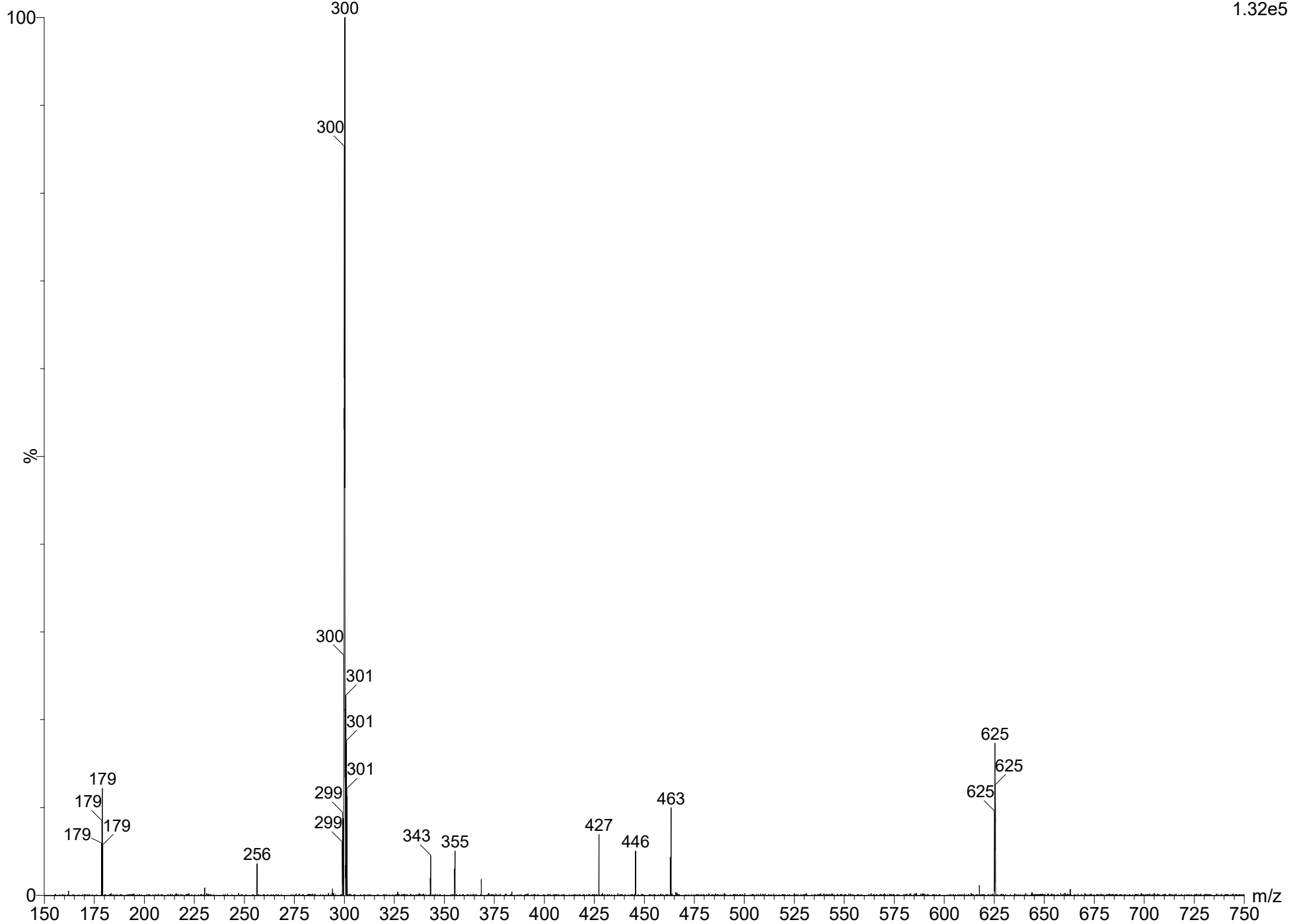

Supplement: Supplementary file 1 [file antioxidants-11-02046-s001.zip › S26-Fragmentation pattern of compound 8.pdf]

orzech\_pylek\_2a 3725 (3.103)

2: Diode Array  
2.244e-1

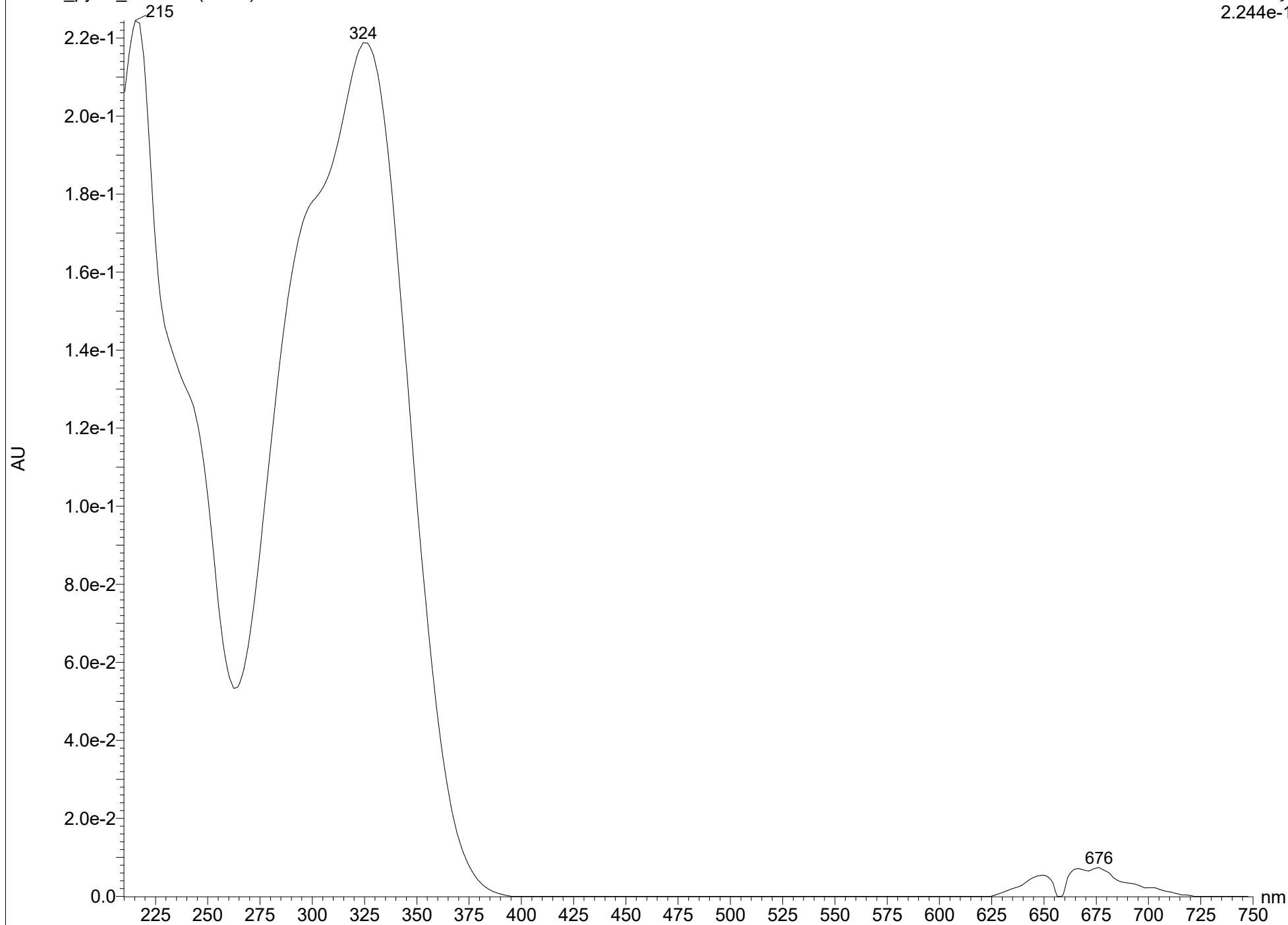

Supplement: Supplementary file 1 [file antioxidants-11-02046-s001.zip › S3-Compound_2_23_UV_Spectra.pdf]

orzech\_pylek\_2 408 (3.476) Cm (407:413)

1: Scan ES-  
7.82e5

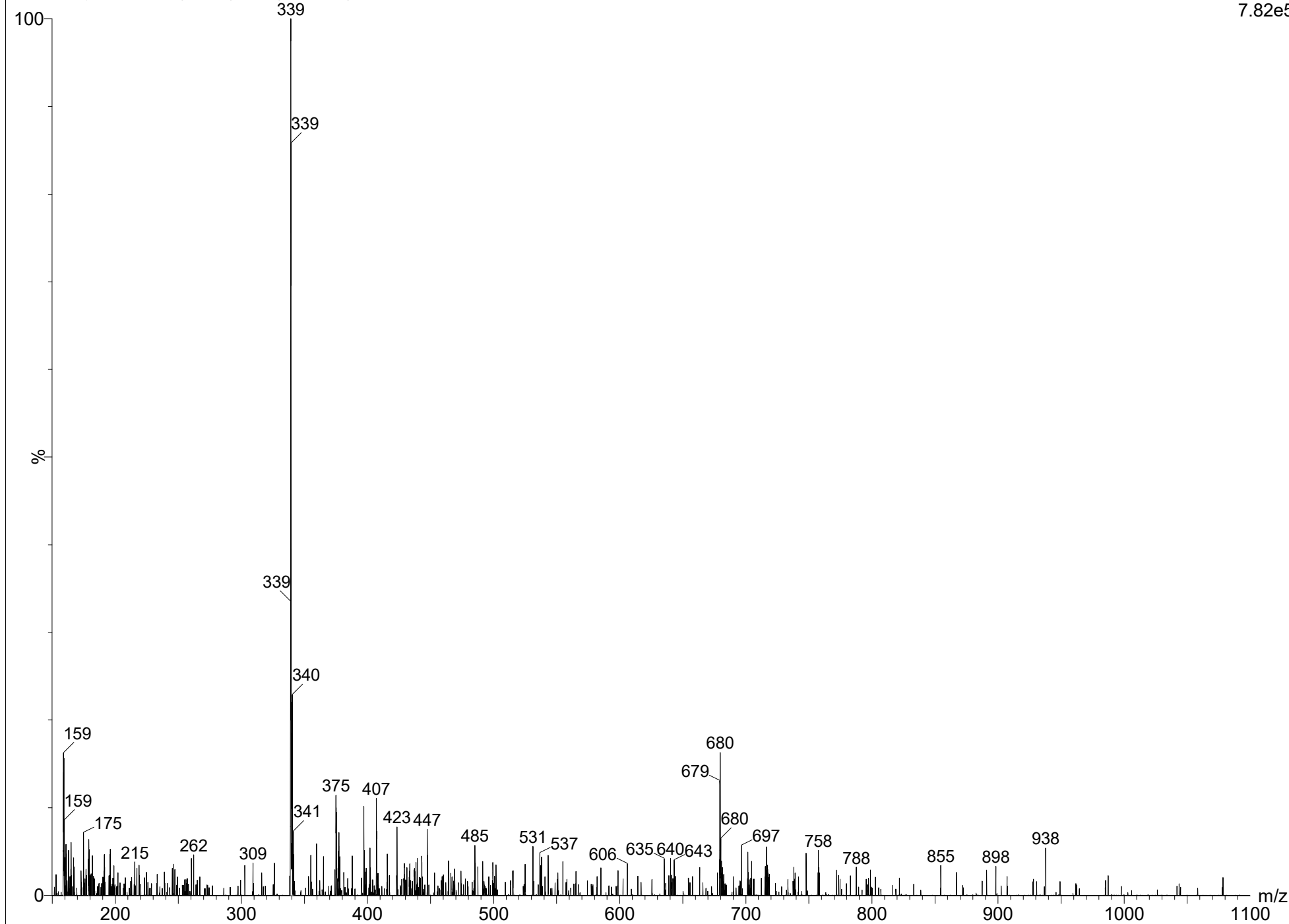

Supplement: Supplementary file 1 [file antioxidants-11-02046-s001.zip › S4-Compound_3_MS_Spectrum.pdf]

orzech\_pylek\_2 423 (3.604) Cm (418:429)

1: Scan ES-  
9.77e4

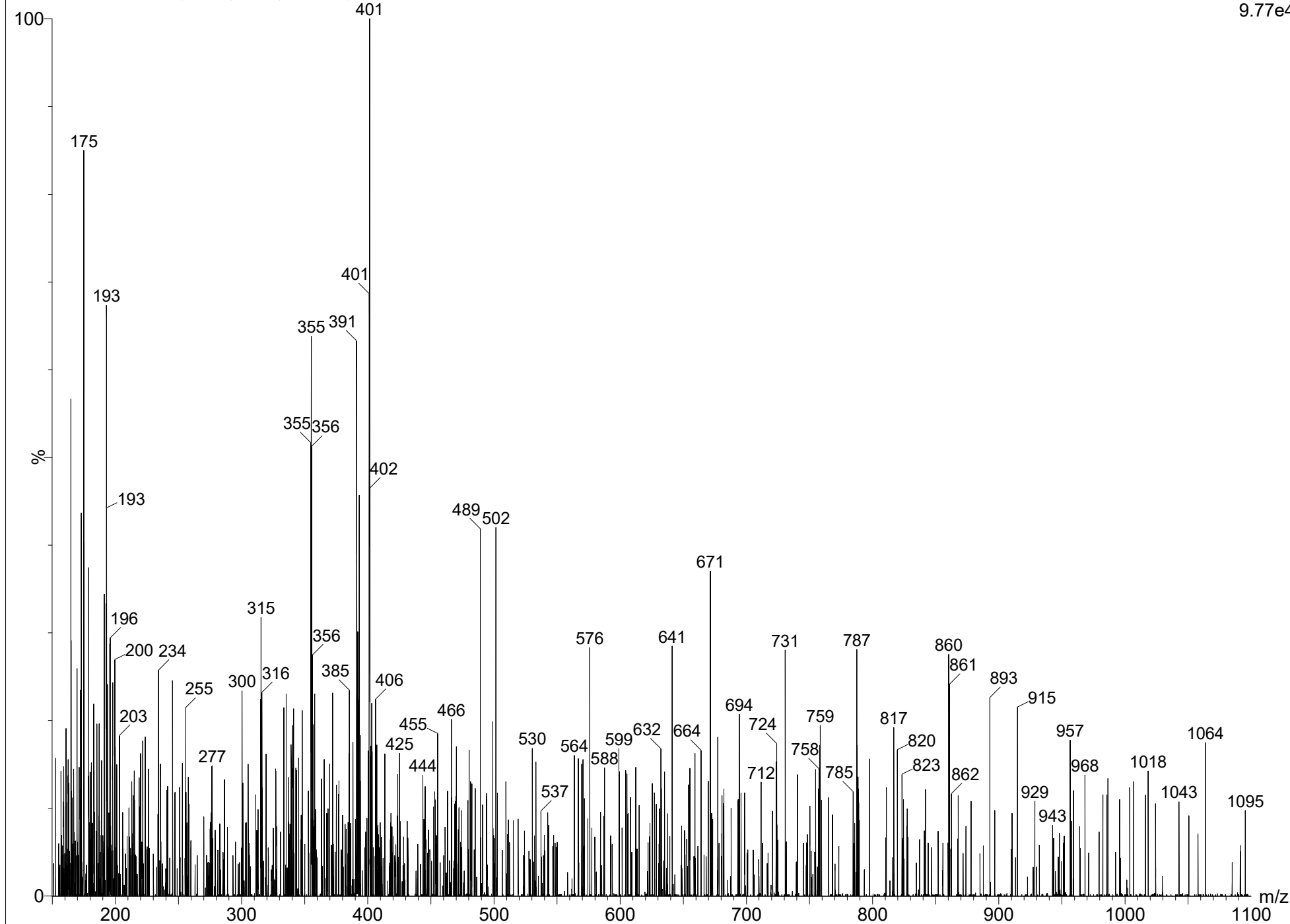

Supplement: Supplementary file 1 [file antioxidants-11-02046-s001.zip › S5-Compound_4_MS_Spectra.pdf]

orzech\_pylek\_2 439 (3.740) Cm (435:444)

1: Scan ES-  
1.27e5

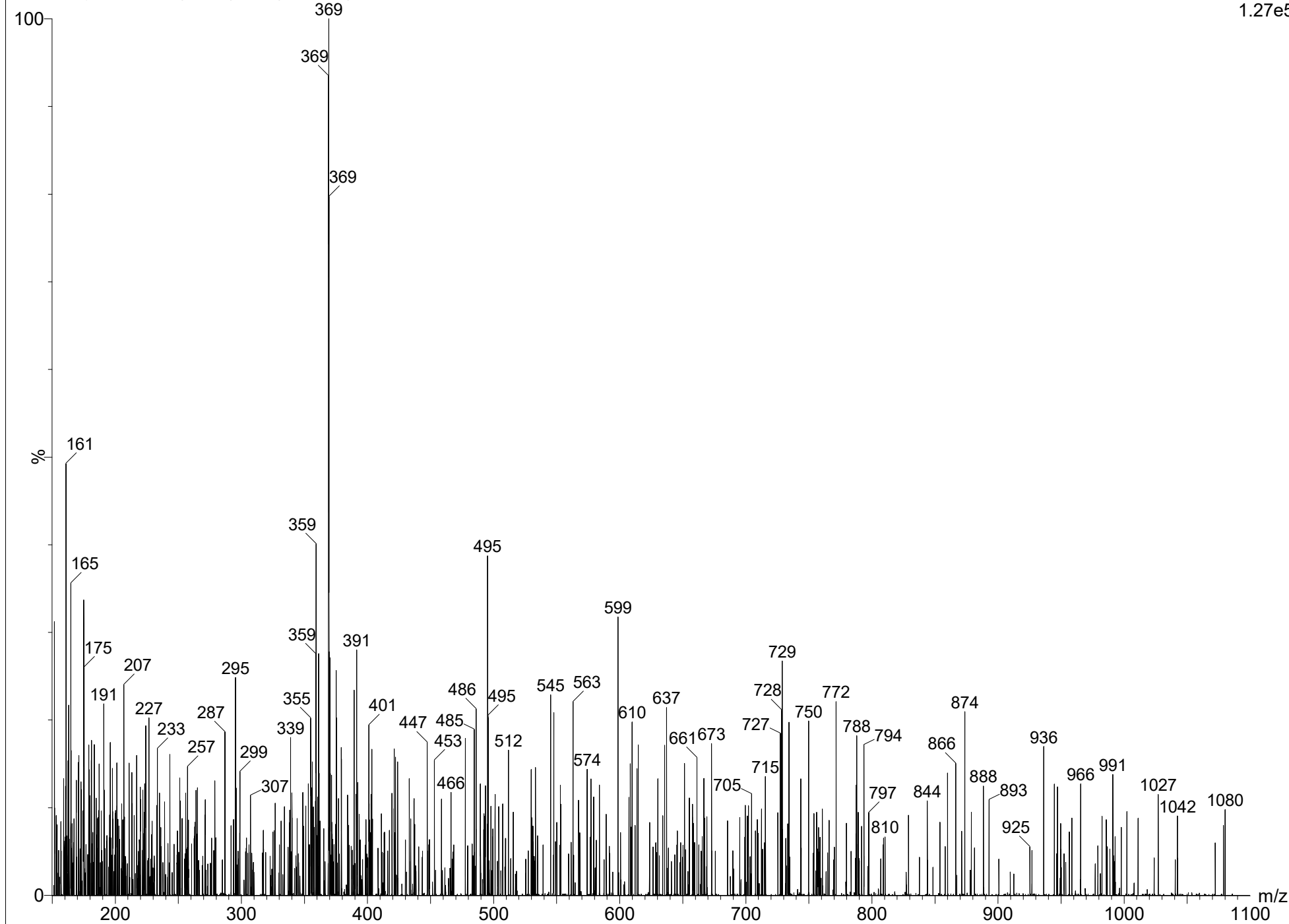

Supplement: Supplementary file 1 [file antioxidants-11-02046-s001.zip › S6-Compound_5_MS_Spectra.pdf]

orzech\_pylek\_2 450 (3.834) Cm (445:453)

1: Scan ES-  
2.05e5

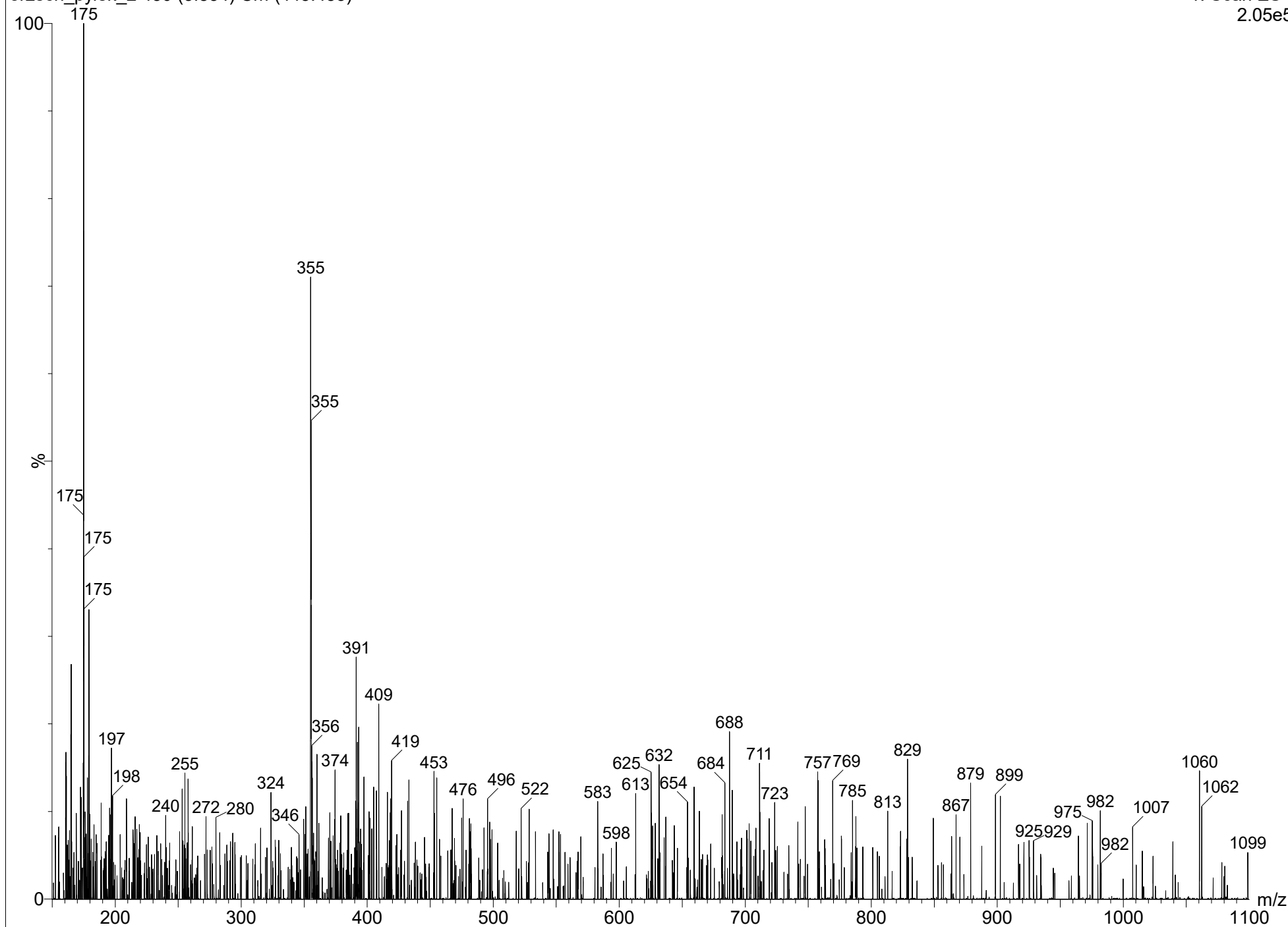

Supplement: Supplementary file 1 [file antioxidants-11-02046-s001.zip › S7-Compound_6_MS_Spectra.pdf]

orzech\_pylek\_2 499 (4.251) Cm (497:503)

1: Scan ES-  
6.69e5

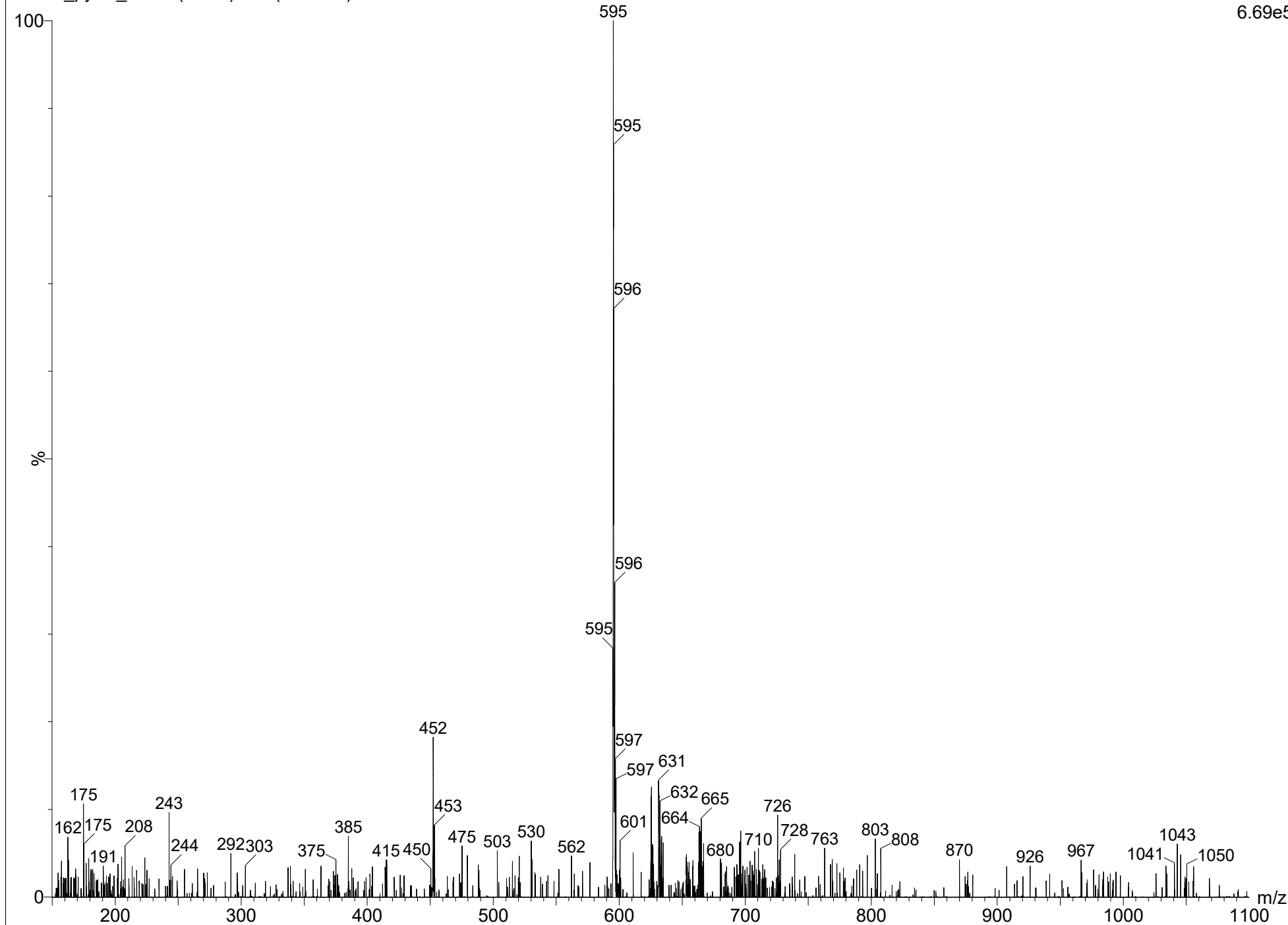

Supplement: Supplementary file 1 [file antioxidants-11-02046-s001.zip › S8-Compound_9_MS_Spectrum.pdf]

orzech\_pylek\_2 505 (4.302) Cm (502:509)

1: Scan ES-  
4.82e5

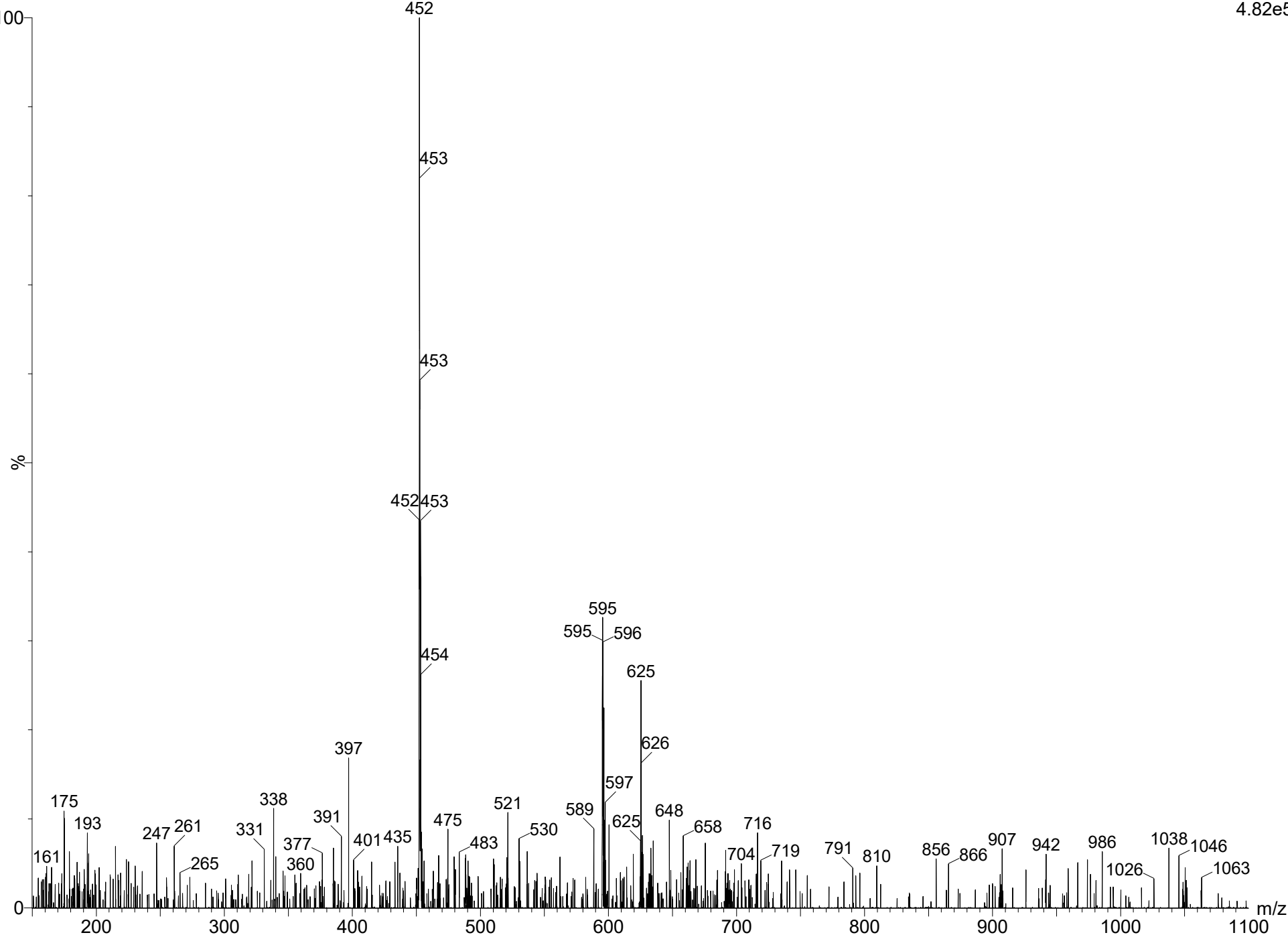

Supplement: Supplementary file 1 [file antioxidants-11-02046-s001.zip › S9-Compound_10_MS_Spectrum.pdf]
